# Chemical Diversity of *Codium bursa* (Olivi) C. Agardh Headspace Compounds, Volatiles, Fatty Acids and Insight into Its Antifungal Activity

**DOI:** 10.3390/molecules24050842

**Published:** 2019-02-27

**Authors:** Igor Jerković, Marina Kranjac, Zvonimir Marijanović, Bojan Šarkanj, Ana-Marija Cikoš, Krunoslav Aladić, Sandra Pedisić, Stela Jokić

**Affiliations:** 1 Faculty of Chemistry and Technology, University of Split, 21000 Split, Croatia; mkranjac@ktf-split.hr (M.K.); zmarijanovic@ktf-split.hr (Z.M.); 2 Department of Food Technology, University Center Koprivnica, University North, Trg dr. Žarka Dolinara 1, 48000 Koprivnica, Croatia; bsarkanj@unin.hr; 3 Faculty of Food Technology, Josip Juraj Strossmayer University of Osijek, 31000 Osijek, Croatia; acikos@ptfos.hr (A.-M.C.); stela.jokic@ptfos.hr (S.J.); 4 Croatian Veterinary Institute, Branch-Veterinary Institute Vinkovci, Josipa Kozarca 24, 32100 Vinkovci, Croatia; k2aladic@gmail.com; 5 Faculty of Food Technology and Biotechnology, University of Zagreb, Pierottijeva 6, 10000 Zagreb, Croatia; spedisic@pbf.hr

**Keywords:** headspace solid-phase microextraction (HS-SPME), distillation, supercritical CO_2_ extraction, gas chromatography and mass spectrometry (GC-MS)

## Abstract

The focus of present study is on *Codium bursa* collected from the Adriatic Sea. *C. bursa* volatiles were identified by gas chromatography and mass spectrometry (GC-FID; GC-MS) after headspace solid-phase microextraction (HS-SPME), hydrodistillation (HD), and supercritical CO_2_ extraction (SC-CO_2_). The headspace composition of dried (HS-D) and fresh (HS-F) *C. bursa* was remarkably different. Dimethyl sulfide, the major HS-F compound was present in HS-D only as a minor constituent and heptadecane percentage was raised in HS-D. The distillate of fresh *C. bursa* contained heptadecane and docosane among the major compounds. After air-drying, a significantly different composition of the volatile oil was obtained with (*E*)-phytol as the predominant compound. It was also found in SC-CO_2_ extract of freeze-dried *C. bursa* (FD-CB) as the major constituent. Loliolide (3.51%) was only identified in SC-CO_2_ extract. Fatty acids were determined from FD-CB after derivatisation as methyl esters by GC-FID. The most dominant acids were palmitic (25.4%), oleic (36.5%), linoleic (11.6%), and stearic (9.0%). FD-CB H_2_O extract exhibited better antifungal effects against *Fusarium* spp., while dimethyl sulfoxide (DMSO) extract was better for the inhibition of *Penicillium expansum, Aspergillus flavus*, and *Rhizophus* spp. The extracts showed relatively good antifungal activity, especially against *P. expansum* (for DMSO extract MIC_50_ was at 50 µg/mL).

## 1. Introduction

Due to their biological and chemical variations, algae have drawn attention as a source of bioactive compounds whose concentration depends on the season, growth conditions, location, and environmental changes [1]. The algae are more frequently represented by three groups (green (*Chlorophyta*), brown (*Phaeophyta*), and red (*Rhodophyta*)). They contain specific bioactive compounds (e.g., brown algae contain specific polyphenols called phlorotannins [2], red algae have been the main producers of halogenated compounds [3]. Green algae have been the least represented in the literature).

Hexadecatrienoic acid is a possible chemotaxonomic marker of the species belonging to the genus *Codium* [4,5] along with clerosterol [6,7,8]. These species also contain long chain fatty acids with palmitic acid as the main saturated fatty acid (SFA) and oleic acid as the most common monounsaturated fatty acid (MUFA) [9]. α-Linolenic acid (C18:3ω3) was the most abundant polyunsaturated fatty acid (PUFA) found in green algae [4]. The investigation of green algae showed that their ω6/ω3 ratio was very preferable for including them in diets with potential beneficial effect for human health [10]. During the growth and adaptation to abiotic stress, algae produce different volatile organic compounds (VOCs) which serve for the communication and interaction with the surrounding environment. Their concentration and composition depend on the environmental conditions, species, and geographical origin, as well as drying process and method used for their extraction [11]. Gressler et al. [12] reviewed about 295 volatile compounds isolated from 31 species of marine macroalgae. Our research group have also been investigating algae VOCs by different isolation techniques. Hydrocarbons were the most common volatile compounds found in algae, followed by alcohols, terpenes, ketones, and aldehydes. Volatile oils, as well as the extracts, from brown and red algae, were tested for the inhibition of several bacteria growths [13,14]. Hydrocarbons were found to be dominant in the volatile oil of *Codium fragile* [15], while long-chain aliphatic alcohols were the major compounds in *Codium tomentosum* [16]. In general, the presence of dimethyl sulfide in the algae contributes to the distinct algae flavor [17].

Different studies report that bioactive secondary metabolites isolated from various green macroalgal species exhibit potential for being used as anti-inflammatory, antioxidant [18], antimicrobial [19] or antigenotoxic [20] molecules. However, the species belonging to the genus *Codium* have been the least investigated for their biological activity among all *Chlorophyceae. C. fragile* has drawn the most attention due to its invasive nature and potential of being utilized for biomedical applications. Surget et al. [21] reported its potential with pro-mineralogenic activity and osteogenic properties. Clerosterol possess great bioactivity potential including the inhibition of human melanoma cell growth [22]. Isolated clerosterol and its derivatives from *Codium arabicum* exhibited cytotoxic activity [23]. *Codium* species were tested for antibacterial activity against pathogenic bacteria. Methanol extracts of *C. dichotomum*, *C. fragile*, *C. Bursa*, and *C. tomentosum* showed significant inhibitory activity against *Staphylococcus aureus*, while *C. bursa* did not inhibit the growth of tested bacteria. Antibacterial activity was influenced by the algal reproductive state and seasonality [24]. Ethanol extracts of *C. bursa* were used against *Escherichia coli* and *Staphylococcus simulans*. All the algal extracts showed important antibacterial activity against investigated bacteria [18]. 

In general, available data on the phytochemical composition and bioactivity of marine macroalgae from the Adriatic sea are limited. Consequently, the focus of this study was on macroalga *Codium bursa* (Olivi) C. Agardh collected from the Adriatic Sea. It belongs to the family Codiaceae and phylum *Chlorophyta* [4], and mostly, it can be found in temperate and subtropical areas [25]. It grows in the range of a few millimeters up to 40 cm in diameter in hollow spherical form. This alga can satisfy its nutrient demands by recycling and preserving nutrient retention within the enclosed water with the presence of a unique microbial heterotrophic community [26]. Thick algal walls enable it to minimize the losses of nutrients which results in low nutrient requirements [27]. *C. bursa* is ranked among the slowest-growing macroalgae because of its low respiration and photosynthetic rates [28]. To the best of our knowledge, this is the first report on its comprehensive headspace, volatile, and less-volatile organic compounds (among them biologically active molecules can be expected). The VOCs of fresh and corresponding air-dried sample of *C. bursa* were investigated to detect the influence of drying on VOCs′ chemical composition determined by hydrodistillation (HD) and headspace solid-phase microextraction (HS-SPME). Freeze-dried sample of *C. bursa* (FD-CB, as a more standardized sample) was further analyzed. It was subjected to supercritical CO_2_ extraction (SC-CO_2_) to determine less-volatile organic compounds that could not be isolated by hydrodistillation (HD) or HS-SPME. In addition, fatty acids’ profile and antifungal activity of FD-CB were also determined for the first time. The goals of present research on *C. bursa* were to: (a) investigate the phytochemical composition of fresh and air-dried *C. bursa* by gas chromatography with flame ionization detector (GC-FID) and with mass spectrometry (GC-MS) after HS-SPME and HD; (b) analyze the less-volatile compounds of SC-CO_2_ extracts of FD-CB by GC-FID and GC-MS; (c) determine fatty acids profile of FD-CB after derivatization as methyl esters by GC-FID; and (d) get an insight into the antifungal activity of FD-CB extracts (with H_2_O and DMSO) against most common mycotoxigenic fungi.

## 2. Results and Discussion

### 2.1. Headspace, Volatile, and Semi-Volatile Organic Compounds

In the present study, two different methods were used to investigate the chemical composition of *C. bursa* volatiles: headspace solid-phase microextraction (HS-SPME) and hydrodistillation (HD). Two fibers of different polarity (divinylbenzene/carboxene/polydimethylsiloxane (DVB/CAR/PDMS) and polydimethylsiloxane/divinylbenzene (PDMS/DVB)) were used for HS-SPME to obtain more complete headspace profiles. Different methods were chosen to obtain a comprehensive profile of the headspace, low and medium volatile compounds. First insight into Table 1 reveals meaningful differences among the headspace and distillate composition that was confirmed by statistical analysis. In addition, the composition of fresh and air-dried *C. bursa* differed notably among the headspace and distillate indicating the strong impact of drying (also confirmed by statistical analysis).

The major headspace compound of fresh *C. bursa* (HS-F) was 2-thiapropane (dimethyl sulfide, DMS) ranging from 36.22% to 56.51%, being more abundant when applying HS-SPME with DVB/CAR/PDMS fiber. It has long been recognized that algae play a highly significant role in the global biogeochemical cycles of sulfur. The key chemical compounds driving these cycles are often low molecular weight and/or volatile compounds. In the case of sulfur, a dominant compound is dimethyl sulfide (DMS), algal osmolyte, which derives from dimethylsulfoniopropionate (DMSP) that has been synthetized and accumulated in a wide range of taxa which occur in diverse ecosystems [29]. DMSP and its breakdown products (e.g., DMS) readily scavenge hydroxyl radicals and other reactive oxygen species and, thus, may serve as an antioxidant system (e.g., for the reduction of oxidative stress), regulated in part by DMSP enzymatic cleavage [30]. DMPS has been found in green algae, and the spatial and temporal distribution of DMSP content in *C. fragile* of the Atlantic coast of Nova Scotia was investigated [31]. Heptadecane was another abundant compound (4.82%; 32.51%) in HS-F, being more abundant when applying HS-SPME with PDMS/DVB fiber. Saturated and olefinic hydrocarbons were already determined in different species of benthic marine algae [32,33]. Heptadecane dominated in red algae but was also abundant in *C. fragile* (89%) after the extraction, chromatographic purification, and GC analysis (before and after hydrogenation) [32]. In general, aliphatic hydrocarbons result from highly endergenic metabolic processes of fatty acids decarboxylation [34]. The results of an incubation of stearic-18-^14^C acid showed an exclusive incorporation of radioactivity into heptadecane indicating direct decarboxylation of stearic acid [34]. Several low-molecular aliphatic aldehydes were present, such as nonanal (3.51%; 2.51%), octanal (0.81%; 0.10%), and decanal (1.01%; 0.42%). They could originate from ω9 MUFAs and also from ω6 PUFAs, such as linoleic acid [35]. Aliphatic lower alcohols oct-1-en-3-ol (1.12%; 9.71%) and octan-1-ol (0.62%; 0.20%) were also found. The biosynthetic pathways of vinylic alcohols in diatoms include the lyase activity and utilize water from the medium, presumably to assist the carbon–carbon bond cleavage and lipoxygenase catalyzes both the oxygenation of arachidonic acid and the lyase reaction leading to the formation of oct-1-en-3-ol [36]. Another abundant compounds in HS-F were aromatic compounds (benzaldehyde (5.21%; 4.73%) and benzyl alcohol (9.31%; 0.20%)) that are known to derive from phenylalanine by shortening of the carbon skeleton side chain by the C_2_-unit, which can potentially occur in the plant tissue *via* either β-oxidative pathway or non-oxidatively [37].

The chemical composition of the headspace of dried *C. bursa* (HS-D) was remarkably different. Namely, the major HS-F headspace compound, DMS, was present in the dried sample only as a minor constituent (3.72%; 3.10%) and its percentage fall of was ca. 15.3 and 11.7 times, respectively (Table 1). That could be the consequence of its low molecular mass and high volatility. However, another DMSP breakdown product, dimethylsulfoxide (1.52%; 2.63%), appeared only in HS-D. On the other hand, the percentage of heptadecane raised in HS-D up to 52.62% (Table 1) presumably as the consequence of DMS loss. In addition, several other saturated hydrocarbons appeared in HS-D (Table 1) with minor percentages (e.g., pentadecane, hexadecane, octadecane or nonadecane) as well as two unsaturated alkenes ((*E*)-heptadec-8-ene and nonadec-1-ene). Carotenoid cleavage products, C_13_-norisoprenoids (that could arise enzymatically [38] or non-enzymatically [39] stimulated by light, oxygen, temperature, and acid hydrolysis), were found only in HS-D: (*E*)-α-ionone (6.40%; 3.02%), (*E*)-β-ionone (1.52%; 1.02%,) and 4-ketoisophorone (0.10%; 0.10%). In addition, other oxygenated aliphatic compounds were found in HS-D (Table 1), such as: hexan-1-ol, octan-2,3-dione, 6-methylhept-5-en-2-one, 2-ethylhept-5-en-2-one or (*E*)-oct-2-enal.

The distillate of fresh *C. bursa* (HD-F) contained as the major compounds higher saturated aliphatic hydrocarbons heptadecane (23.44%) and docosane (13.90%) (Table 1). Higher heptadecane abundance was expected according to HS-SPME results. Docosane was identified previously in *C. fragile* after steam distillation of the dichloromethane extract [40] and with minor percentages (1.14%) in *C. fragile* essential oil [15]. Hydrocarbons dominated [15] in the essential oil isolated from freeze-dried samples of *C. fragile*, such as tricosane (11.88%), 2,2,4-trimethyl-1,3-dioxolane, (8.53%), hexadecane (4.07%), eicosane (3.83%), and tetracosane (3.48%). Other abundant compounds (Table 1) were diisooctyl phtalate (13.30%) and dibutyl phtalate (9.80%). It has been shown by analyzing the natural abundance ^14^C content of the isolated compounds and industrial products that dialkyl phthalates can be naturally produced by algae [41]. (*E*)-Phytol was identified in the essential oil with 3.31% along with its derivative phytone (1.61%). The presence of linear diterpene alcohol *trans*-phytol and its acetate derivatives were previously reported in the extract of *C. fragile* [8].

After air-drying *C. bursa, a* significantly different chemical composition of the volatile oil was obtained (HD-D). Namely, (*E*)-phytol predominated (58.42%), and its percentage raised ca. 17.7 times in comparison to HD-F, probably as the consequence of chlorophyll breakdown [42] and *trans*-phytol was also found in the extract of *C. fragile* [8]. In addition, the percentage of phytone raised ca. 3.5 times compared to the fresh sample. Oxidation of phytol moiety of chlorophyll could lead, among others, to the methylated long chain fatty acid ketone-hexahydroxyfarnesyl acetone (6,10,14-trimethylpentadecan-2-one, phytone) that was found in the oil from the dried sample at 5.91%. Another significant change in HD-D was a significant decrease (9.8 times for dibutyl phtalate) or disappearance (diisooctyl phtalate) of alkyl phthalates (Table 1). Benzyl alcohol percentage also dropped from 18.02% to 0.10% which is probably due to its evaporation.

The major compound of SC-CO_2_ extract of FD-CB was (*E*)-phytol (42.30%) accompanied by structurally related neophytadiene (3.20%). Other dominant aliphatic compounds were hexadecanoic acid (17.51%), heptadecane (7.20%), hexadecan-1-ol (3.10%), and (*Z*)-octadec-9-enoic acid (3.02%). The composition of SC-CO_2_ extract was partially similar to HD-D (Table 1, VI, and VII). However, monoterpenoid hydroxylactone loliolide (3.51%) was only identified in SC-CO_2_ extract. Among green algae, it was found previously in *Codium divaricatum* Holmes from which it was isolated by ethanol extraction from dried algae [43]. Despite a simple structure, loliolide showed a broad spectrum of biological activity [43,44]: germination inhibitory activity, anti-repellent activity, immunosuppressive activity, as well as growth inhibitory activity against human nasopharynx carcinoma (KB), and murine lymphocytic leukemia (P-388).

### 2.2. Fatty Acids Composition

The species belonging to the genus Codium collected from different locations were investigated for their fatty acids content [45,46,47]. The results of of the present study revealed a total of 19 fatty acids (Table 2) in FD-CB.

The main fatty acids of FD-CB were oleic acid (C18:1n9), as the dominant, followed by palmitic acid (C16:0), linoleic acid (C18:2n6), and stearic acid (C18:0). The total content of unsaturated fatty acids was higher than saturated reaching 57.68% (including both MUFAs (41.73%) and PUFAs (15.95%)). Generally, for green algae it is a characteristic that the amount of unsaturated fatty acids is higher than saturated fatty acids [47]. Palmitic acid (C16:0) was the main SFA which is in agreement with previous studies [5,45]. Oleic acid (C18:0) with 36.54% was found as the main MUFA, while linoleic acid (C18:2n6*c*) was the dominant PUFA with 11.62%. Goecke et al. [4] and Banaimoon [48] showed that linolenic acid was the dominant PUFA in *Codium* species. PUFAs are of great importance for human health due to their activities related to reducing the risk of heart diseases, atherosclerosis, and thrombosis [49]. In the present study, linolenic acid was present in both of the forms, α-linolenic (ω3; C18:3n3) with higher abundance and γ-linolenic (ω6; C18:3n6). ω6 Fatty acids were present at a higher percentage than ω3 fatty acids (Table 2). Although several authors [4,5,10] detected hexadecatrienoic (C16:3) acid in *Codium* species indicating it as chemotaxonomic marker, in the present research C16:3 was not detected in *C. bursa*. Yazici et al. [47] also reported that hexadecatrienoic acid was not detected in *C. fragile*. A possible explanation is that the fatty acid content is influenced by geographical location and environment conditions [46]. It must be taken into consideration that the fatty acid composition can vary among different species within the same genus and also within the same species which were collected at different locations in the same season. Moreover, fresh algal samples gave a higher amount of PUFAs than dried samples which can be explained by the susceptibility of PUFAs to oxidation processes [5].

### 2.3. Antifungal Activity

The experiment aimed to gain insight into the antifungal activity of two extracts of FD-CB, not to investigate the extracts chemical composition or to identify the compounds responsible for the noted activity. Two solvents of different polarity (H_2_O and dimethyl sulfoxide (DMSO)) were used for the extraction of FD-CB. The antifungal activity of the extracts was determined against the most common and problematic fungal species with regard to regulated and emerging mycotoxin production. The obtained results are given in Table 3. The extracts exhibited a relatively low antifungal effect to most common cereals associated mycotoxigenic fungi (*Alternaria alternata*, *Aspergillus flavus*, *Aspergillus ochraceus*, *Fusarium graminearum*, and *Fusarium verticillioides*), and relatively good antifungal effect (MIC_50_ at 50 µg/mL DMSO extract) to fruit associated storage fungi *Penicillium expansum*. When comparing both extracts, there is a significant difference in the antifungal effect suggesting that different components or their concentrations were extracted by two solvents. *Fusarium* spp. was more susceptible to high concentrations of H_2_O extracts, while *P. expansum*, *A. flavus*, and *Rhizophus* spp. were more susceptible to DMSO extracts, although detected MIC_50_ concentrations were several orders of magnitude higher compared with the reference standards (*Amphotericin B*, *Itraconazole*, *Posaconazole*, and *Voriconazole*) [50]. On the other hand, increased growth of several fungal species was also noted, and, therefore, a new parameter was added—growth-inducing concentration (GIC_50_). The new parameter also showed at which concentrations the tested extracts induce the growth (based on the optical density at 405 nm) for at least 50% compared with the control. Based on these calculations some of tested extracts on several fungal species did not show inhibitory properties but rather growth inducing properties, while for other fungal strains there was concentration dependent change from MIC_50_ to GIC_50_. Interestingly, DMSO extract showed the antifungal activity against *Rhizophus* spp. at 5000 µg/mL while H_2_O extract at the same concentration showed growth inducing properties. A similar trend was observed for *P. expansum* where DMSO extract showed excellent antifungal properties, and on the other hand, H_2_O extract exhibited great growth-inducing potential. Both extracts did not show growth-inducing effect against *Fusarium* spp. *A. ochraceus* was only fungi that exhibited susceptibility towards H_2_O extract, and growth-inducing effect towards DMSO extract that was noticeable only when high concentrations were used (5000 µg/mL). *A. flavus* showed great concentration-dependent response to the usage of FD-CB extracts. While the high amount of DMSO extract exhibited antifungal properties (5000 µg/mL), at low concentrations (5 and 50 µg/mL) DMSO extract showed growth inducing properties. Similarly, H_2_O extract at 5 µg/mL increased the growth of the fungi indicating possible hormetic effect. Further characterization of the active compounds in the extracts is in progress.

To the best of the authors’ knowledge, this is the first published paper on the antifungal activity of *C. bursa* against main mycotoxin-producing fungi: *A. alternata*, *A. flavus*, *A. ochraceus*, *F. graminearum*, *F. verticillioides*, *P. expansum*, and food fermenting *Rhiziohus* spp. Therefore, it is possible in discussion only to mention previously observed biological activities of other *Codium* species. Thus, Yang et al. [51] isolated *A. flavus* from the surface of the edible green algae *C. fragile* from Korea. The alga induced the growth of *A. flavus* on its surface, indicating that there is a growth-inducing component. Other published data on *Codium* spp. showed various levels of bactericidal effects. Koz et al. [15] detected the antimicrobial activity of different extracts of *C. fragile*. The volatile components inhibited the growth of *Bacillus subtilis*, and *B. cereus*; the methanol, dichloromethane and hexane extracts inhibited the growth of *B. subtilis*, *Staphylococcus aureus* (methicillin-oxacillin resistant), *Klebsiella aerogenes*, and *Escherichia coli* at 0.25 and/or 0.50 mg/disc. In the minimum inhibitory concentration (MIC) testing, hexane extracts showed the best results inhibiting most of the tested bacterial growth (*B. subtilis*, *B. cereus*, *K. aerogenes*, *E. coli*, *E. coli* (O157:H7), *Pseudomonas aeruginosa*, and *Proteus vulgaris*, at the lowest concentrations (from <50 µg/mL to 1000 µg/mL). The methanol extract showed higher MIC values for gram-positive bacteria *B. subtilis, B. cereus, S. epidermidis*, and *S. aureus*, and gram-negative bacteria *P. aeruginosa*, and *P. vulgaris* at 250 to 500 µg/mL. The dichloromethane extracts did not show any activity in MIC test, and the essential oil from algae showed great bactericidal concentrations but only against tested gram-positive strains (*B. subtilis, B. cereus, S. epidermidis*, and *S. aureus*) at <50 µg/mL. They also tested all extracts and essential oils against fungi *Candida albicans*, but no significant growth inhibition was detected [15]. Ballesteros et al. [52] documented a high antifungal activity of the methanol/toluene (3:1 *v*/*v*) *C. bursa* extracts on different tested fungi (*C. albicans* and *Aspergillus niger*).

## 3. Materials and Methods 

### 3.1. Chemicals

The industry fatty acid esters (FAME) mix 37 standard for fatty acids analysis was purchased from Bellefonte, PA (USA). The purity of CO_2_ used for SC-CO_2_ extraction was 99.97% (*w*/*w*) (Messer, Osijek, Croatia). All other chemicals and reagents were of analytical reagent grade and obtained from Sigma–Aldrich (St. Louis, MO, USA) and 3-(*N*-morpholino)propanesulfonic acid was purchased from Sigma–Aldrich (Chemie GmbH, Taufkirchen, Germany).

### 3.2. Marine Alga Codium bursa

Green alga *Codium bursa* (Olivi) C. Agardh was collected from the middle part of the Adriatic Sea at Iški Mrtovnjak island, in May 2018 (44°00′36″ N; 15°10′36″ E). The alga was collected from a depth of 10 to 15 m and the sea water was collected from the same depth (directly into the plastic bag where the collected alga was placed). The alga which was placed in an airtight plastic bag containing surrounding seawater was immediately transported to the laboratory.

#### Preparation of *C. bursa* for Further Analysis

**Figure 1:**
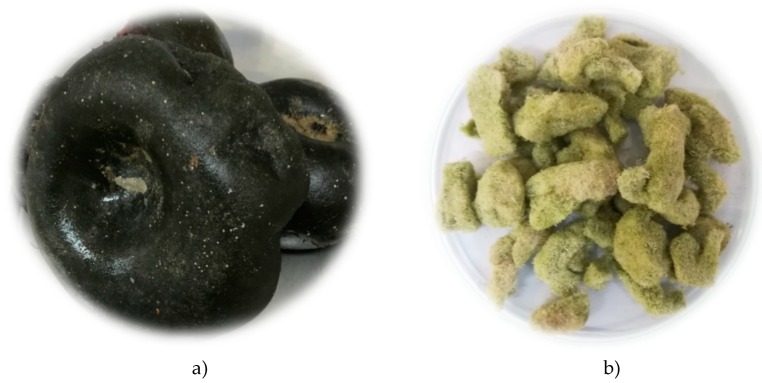
*Codium bursa* (Olivi) C. Agardh: (**a**) fresh sample (F-CB), (**b**) freeze-dried sample (FD-CB).

Three different preparation of *C. bursa* were performed producing three samples:

1. Before HS-SPME and HD, fresh *C. bursa* (50 g; F-CB; Figure 1a) was taken out of the bag, cut into small pieces with laboratory knife, and the excess of seawater was removed by placing the pieces between filter paper layers for 2 min (the seawater was not removed completely) as was done in our previous research [53].
2. The mass of 50 g of fresh *C. bursa* was cut as described above and dried at room temperature in the dark for 10 days, and the air-dried sample was obtained (D-CB).
3. Fresh *C. Bursa* (500 g) was washed five times in water and twice in deionized water. For the freeze-drying experiment the samples were cut in slices (from 5 to 10 mm) and frozen at −60 °C for 24 h in an ultra-low freezer. Five trays of frozen samples were placed in a laboratory freeze dryer (CoolSafe PRO, Labogene, Denmark). The freeze drying process was performed for 24 h under high vacuum (0.13–0.55 hPa) with primary and secondary drying temperatures of −30 °C and 20 °C, respectively. Freeze-dried samples (Figure 1b; FD-CB) were further used for determination of antifungal activity and fatty acids content as well as for supercritical CO_2_ extraction (SC-CO_2_).

### 3.3. Headspace Solid-Phase Microextraction (HS-SPME)

The headspace extraction was performed by manual SPME holder equipped with polydimethylsiloxane/divinylbenzene (PDMS/DVB) fiber and divinylbenzene/carboxene/ polydimethylsiloxane (DVB/CAR/PDMS) fiber obtained from Supelco Co. (Bellefonte, PA, USA). Each fibre was conditioned before the extraction (according to Supelco Co. instructions). For HS-SPME, previously prepared samples of fresh (1 g; F-CB) and dried *C. bursa* (0.7 g; D-CB) were placed separately in 5 mL glass vials and hermetically sealed with polytetrafluorethylene (PTFE)/silicone septa. The vials were maintained in a water bath at 60 °C during the equilibration (15 min) and extraction (45 min). After sampling, the SPME fiber was withdrawn into the needle, removed from the vial, and inserted into the injector (250 °C) of the GC-FID and GC-MS for 6 min. The extracted volatiles were thermally desorbed directly to the GC column. HS-SPME of F-CB was performed within 24 h after the collection. HS-SPME was done in duplicate.

### 3.4. Hydrodistillation (HD)

Hydrodistillation was performed in modified Clevenger apparatus for 2 h with the use of 1 mL of solvent trap (pentane:diethyl ether, 1:2 *v*/*v*). The prepared samples of fresh (20 g; F-CB) and dried *C. bursa* (10 g; D-CB) were used separately for the hydrodistillation. HD of F-CB was performed within 24 h after the collection. The hydrodistillate in solvent trap was removed with the pipette, passed through the layer of MgSO_4_ in small glass funnel and carefully evaporated by the slow flow of nitrogen until the volume of 0.2 mL. HD was performed in duplicate. 1 μL was used for GC-FID and GC-MS analysis.

### 3.5. Supercritical CO_2_ Extraction (SC-CO_2_)

SC-CO_2_ extraction was performed in a supercritical fluid extraction (SFE) system explained in detail previously [54]. The grounded freeze-dried sample of *C. bursa* L. (30 g; FD-CB) was placed into the extractor vessel and the extraction was performed 90 min at temperature of 40 °C and pressure of 300 bar. Dynamic extraction mode for SFE was used where SC-CO_2_ continuously passed through the sample matrix. The obtained extract was diluted with hexane and diethyl ether (1:2 *v*/*v*) and 1 μL was used for GC-FID and GC-MS analysis.

### 3.6. Gas Chromatography and Mass Spectrometry Analysis of VOCs

The GC-FID analyses of volatiles were carried out with an Agilent Technologies (Palo Alto, CA, USA) gas chromatograph model 7890A equipped with a flame ionization detector (FID) and a HP-5MS capillary column (5% phenyl-methylpolysiloxane, Agilent J and W). The GC-MS analyses were performed on an Agilent Technologies (Palo Alto, CA, USA) gas chromatograph model 7890A equipped with a mass selective detector (MSD) model 5977E (Agilent Technologies) and HP-5MS capillary column, under the same conditions as for the GC-FID analysis. The GC conditions and the detail procedure are described in our previously published paper [53]. The identification of the compounds was based on the comparison of their retention indices (RI), determined relative to the retention times of C_9_–C_25_ homologous series of *n*-alkanes with those reported in the literature and on the comparison of their mass spectra with available authentic compounds or with the mass spectra listed in Wiley 9 (Wiley, New York, NY, USA) and NIST 14 (D-Gaithersburg) mass spectral libraries. The percentage composition of the samples was computed from the GC peak areas using the normalization method (without correction factors). The average component percentages in Table 1 were calculated from duplicate GC-FID and GC-MS analyses. One-way analysis of variance (ANOVA) and multiple comparisons (Duncan’s *post-hoc* test) were used to evaluate the significant difference of the data at *p* < 0.05 (Table 1.).

### 3.7. GC-FID Analysis of Fatty Acids

Fatty acid methyl esters were prepared according to HRN EN ISO 12966-2:2011 standard [55]. Prepared fatty acid methyl esters were analyzed by gas chromatography according to HRN EN ISO 12966-4:2015 [56]. Gas chromatograph 7890A (Agilent Technologies, Lake Forest, CA, USA) with a capillary column ZB-WAX 25 m long with a diameter of 0.25 mm and the thickness of the stationary phase 0.25 microns (Phenomenex, Torrance, CA, USA), a split–splitless injector (temperature 260 °C) and a flame ionization detector (temperature 280 °C) was used. A sample (5 µL) was injected with a split ratio of 1:40. Start column temperature was 60 °C with holding time for 2 min. The oven temperature was increased at the rate of 13 °C/min to 150 °C, then at the rate of 2 °C/min was heated to 240 °C. The carrier gas was helium (99.9999%) at a constant flow rate of 3 mL/min. The hydrogen flow was 70 mL/min, air flow was 450 mL/min, and the makeup gas flow (nitrogen) was 15 mL/min. Fatty acid methyl esters were identified by comparison with retention times of 37 fatty acid methyl ester standard compounds analyzed at the same conditions. The result is expressed as a percentage (%) of individual fatty acids to total fatty acids. The detection limit of the method was 0.1%.

### 3.8. Antifungal Testing

Antifungal testing was performed in accordance with the guidelines (document M38-A) [57] with modifications described by Šarkanj et al. [58]. In this study the most important mycotoxin-producing species [59] were chosen as tested fungi. *Aspergillus flavus* (NRRL 3251)*, Aspergillus ochraceus* (CBS 589.68)*, Fusarium graminearum* (CBS 110.250)*, Fusarium verticillioides* (119.825)**, and *Penicillium expansum* (CBS 164.59) were used as producers of regulated mycotoxins (aflatoxins, ochratoxin A, deoxynivalenol, T-2 and HT-2 toxins, fumonisins, zearalenon, patulin, and citrinin). In addition, several species that are important producers of emerging mycotoxins were also used, such as *Alternaria alternata* (wild type) and *Rhizophus* spp. (wild type). For the inoculum preparation, the fungal spores were harvested from 7 days old potato dextrose agar (PDA) slants grown at 25 °C in the dark (except for *Fusarium* spp. where the mung bean agar (MBA) was used instead of PDA as a better alternative for sporulation). The number of the spores for inoculation was adjusted to 10^6^ CFU/mL by using Haemocytometer. For the antifungal susceptibility testing the RPMI 1640 medium buffered with 0.164 M MOPS (3-(*N*-morpholino)propanesulfonic acid) at pH 7.0 was sterilized by filtration through 0.22 µm filter.

The ground, freeze-dried sample of *C. bursa* L. (1 g; FD-CB) was used for ultrasound-assisted extraction with water (H_2_O) and dimethyl sulfoxide (DMSO) as a solvent using an ultrasound bath with temperature control at 37 kHz and the ultrasonic power of 50 W (Elma, Elmasonic P 70 H, Elma Schmidbauer GmbH, Gottlieb–Daimler, Singen, Germany). The obtained extracts (500 mg/10 mL) were sterilized by filtration and diluted to tested concentrations (5000, 500, 50, and 5 µg/mL) in sterile buffered RPMI 1640 media. The plates were incubated at 35 °C in the dark during 72 h (to ensure stable growth of all tested fungi). After growth, the plates were read on a microplate reader (Azure Ao Absorbance microplate reader, Azure biosystems, Dublin CA, USA) at 405 nm. The Minimal inhibitory concentration for 50% cells (MIC_50_) was defined as the lowest concentration reducing the optical density by 50% at 405 nm compared to growth control.

During the experiment growth, increasement was also noted in some cases compared to control and, therefore, growth inducing concentration (GIC_50_) for at least 50% of the cells was defined as the tested extract concentration where optical density was at least 50% higher at 405 nm compared to growth control.

## 4. Conclusions

The present research contributes toward better chemical characterization of marine green alga *Codium bursa* (Olivi) C. Agardh from the Adriatic Sea. According to the obtained results, the composition of volatile compounds differed significantly considering applied extraction methods. The major headspace compound of HS-F was 2-thiapropane (DMS), while in HS-D it was present as a minor constituent. (*E*)-phytol was obtained in HD-D and in the SC-CO_2_ extract as a major constituent, and its predominance was probably originated after chlorophyll breakdown. HD-F was abundant in higher aliphatic hydrocarbons, such as heptadecane and docosane, probably as the consequence of DMS loss. The presence of loliolide was determined only in SC-CO_2_ extract. Therefore, the use of different methods enabled obtaining full chemical profiles of the headspace, volatile, and semi-volatile compounds and combined used of all of them can be recommended for chemical profiling of *C. bursa* (the single-use one method would result with incomplete profiles that are clearly visible even with HS-SPME when two fibers revealed significantly different results). The obtained results for fatty acids of *C. bursa* showed a predominance of unsaturated fatty acids over the saturated fatty acids. Oleic acid was found as the main fatty acid, followed by palmitic, linoleic, and stearic acid.

There is a significant difference in the activity against tested fungal species when H_2_O and DMSO extracts were used that could be the influence of different solvent polarity to the compounds’ extraction. H_2_O extract exhibited better antifungal effects against *Fusarium* spp., while DMSO extract was better for the inhibition of *P. expansum, A. flavus*, and *Rhizophus* spp. On the other hand, the extracts showed growth inducing properties for some fungal species. H_2_O extract showed growth inducing properties when tested on *Rhizopus* spp. and *P. expansum*, while DMSO extract showed inhibition properties. In general, it can be concluded that the extracts of *C. bursa* showed relatively good antifungal activity, especially against *P. expansum*.

This research contributes to a better characterization of marine macroalgae from the Adriatic Sea and provides information about marine algal biodiversity. This alga can be of great interest for future investigations considering its phytochemical profile, as well as biologically active compounds providing observed antifungal activity.

## Figures and Tables

**Table 1:** The volatile compounds from *Codium bursa* isolated by headspace solid-phase microextraction (HS-SPME), hydrodistillation (HD), supercritical CO_2_ extraction (SC-CO_2_) and analysed by gas chromatography and mass spectrometry (GC-FID and GC-MS).

No	Compound	RI	RI_L_	Area Percentages (%)						
				I ± SD	II ± SD	III ± SD	IV ± SD	V ± SD	VI ± SD	VII ± SD
1.	2-Thiapropane (DMS) ^S^	<900	521	56.51 ± 2.45 ^a^	3.72 ± 0.10 ^b^	36.22 ± 1.58 ^c^	3.10 ± 0.10 ^b^	-	-	-
2.	Butanal ^S^	<900	598	-	-	-	-	-	-	-
3.	Pentan-1-ol ^S^	<900	768	-	1.02 ± 0.10 ^a^	-	-	-	-	-
4.	Hexanal ^S^	<900	801	1.44 ± 0.15 ^a^	1.41 ± 0.11 ^a^	0.20 ± 0.01 ^b^	0.71 ± 0.02 ^b^	-	-	-
5.	Dimethyl-sulfoxide ^S^	<900	/	-	1.52 ± 0.14 ^a^	-	2.63 ± 0.10 ^b^	-	-	-
6.	Ethylbenzene ^S^	<900	858	-	2.23 ± 0.08 ^a^	-	0.42 ± 0.01 ^b^	-	-	-
7.	Hexan-1-ol ^S^	<900	867	0.62 ± 0.01 ^a^	-	-	-	-	-	-
8.	Nonane ^S^	900	900	-	-	-	-	-	0.10 ± 0.01 ^a^	-
9.	α-Pinene ^S^	940	940	1.43 ± 0.09 ^a^	-	-	0.32 ± 0.01 ^b^	0.81 ± 0.02 ^a^	-	-
10.	Benzaldehyde ^S^	965	964	5.21 ± 0.15 ^a^	6.14 ± 0.11 ^b^	4.73 ± 0.09 ^a^	1.42 ± 0.02 ^c^	1.40 ± 0.01 ^c^	1.41 ± 0.03 ^c^	-
11.	Oct-1-en-3-one ^S^	981	980	-	-	-	0.10 ± 0.01 ^a^	-	-	-
12.	Oct-1-en-3-ol ^S^	982	982	1.12 ± 0.14 ^a^	2.61 ± 0.10 ^b^	9.71 ± 0.18 ^c^	0.81 ± 0.02 ^a^	-	-	-
13.	Octan-2,3-dione	985	986	-	0.80 ± 0.03 ^a^	-	0.41 ± 0.01 ^a^	-	-	-
14.	6-Methyl-hept-5-en-2-one ^S^	988	988	-	1.42 ± 0.08 ^a^	-	0.41 ± 0.02 ^b^	-	-	-
15.	2-Pentylfuran ^S^	992	991	-	0.30 ± 0.01 ^a^	-	-	-	0.60 ± 0.02 ^a^	-
16.	Octanal ^S^	1003	1003	0.81 ± 0.02 ^a^	0.32 ± 0.01 ^a^	0.10 ± 0.01 ^a^	0.40 ± 0.01 ^a^	-	-	-
17.	δ-3-Carene ^S^	1013	1013	-	0.10 ± 0.01 ^a^	-	0.30 ± 0.01 ^a^	-	-	-
18.	*p*-Cymene ^S^	1031	1031	0.51 ± 0.02 ^a^	-	-	0.10 ± 0.01 ^a^	-	-	-
19.	2-Ethyl-hexan-1-ol ^S^	1032	1031	-	0.40 ± 0.01 ^a^	-	0.72 ± 0.01 ^a^	-	-	-
20.	Limonene ^S^	1035	1035	2.20 ± 0.09 ^a^	-	-	-	-	-	-
21.	Benzyl alcohol ^S^	1037	1037	9.31 ± 0.30 ^a^	3.42 ± 0.09 ^b^	0.20 ± 0.01 ^c^	5.40 ± 0.03 ^b^	18.02 ± 1.04 ^d^	0.10 ± 0.01 ^c^	-
22.	(*E*)-Oct-2-enal ^S^	1061	1062	-	0.80 ± 0.01 ^a^	-	0.11 ± 0.01 ^a^	-	-	-
23.	Octan-1-ol ^S^	1074	1074	0.62 ± 0.03 ^a^	0.71 ± 0.04 ^a^	0.20 ± 0.01 ^a^	0.30 ± 0.01 ^a^	-	-	-
24.	Nonanal ^S^	1103	1102	3.51 ± 0.15 ^a^	1.00 ± 0.05 ^b^	2.51 ± 0.14 ^c^	1.40 ± 0.05 ^b^	-	0.62 ± 0.01 ^b^	-
25.	4-Keto-isophorone ^S^	1147	1147	-	0.10 ± 0.01 ^a^	-	0.10 ± 0.01 ^a^	-	-	-
26.	6-[(*Z*)-1-Butenyl]-cyclohepta-1,4-diene (Dictyo-pterene D)	1158	/	-	-	-	0.41 ± 0.01 ^a^	-	-	-
27.	6-Butyl-cyclohepta-1,4-diene (Dictyo-pterene C)	1174	/	-	-	-	0.40 ± 0.01 ^a^	-	-	-
28.	Decanal ^S^	1206	1206	1.01 ± 0.03 ^a^	0.42 ± 0.01 ^a^	0.43 ± 0.01 ^a^	0.80 ± 0.02 ^a^	-	-	-
29.	2-Phenoxy-ethanol ^S^	1215	1213	-	-	-	-	-	-	6.02 ± 0.16 ^a^
30.	β-Cyclocitral ^S^	1222	1223	-	0.50 ± 0.01 ^a^	-	0.42 ± 0.01 ^a^	-	-	-
31.	Farnesane ^S^	1376	1376	-	0.42 ± 0.02 ^a^	-	0.71 ± 0.03 ^a^	-	-	-
32.	Tetradecane ^S^	1400	1400	-	-	-	0.30 ± 0.01 ^a^	-	0.71 ± 0.01 ^a^	-
33.	Dodecanal ^S^	1409	1411	-	0.31 ± 0.01 ^a^	-	0.10 ± 0.01 ^a^	-	-	-
34.	(*E*)-α-Ionone ^S^	1428	1429	-	6.40 ± 0.30 ^a^	-	3.02 ± 0.19 ^b^	-	2.22 ± 0.08 ^b^	-
35.	Geranyl acetone ^S^	1454	1454	-	0.10 ± 0.01^a^	-	0.31 ± 0.01^a^	-	-	-
36.	β-Selinene ^S^	1462	1464	-	0.50 ± 0.02 ^a^	-	-	-	-	-
37.	Ledene ^S^	1472	1473	-	0.71 ± 0.01 ^a^	-	-	-	-	-
38.	Dodecan-1-ol ^S^	1477	1476	-	0.42 ± 0.01 ^a^	-	-	-	-	-
39.	ar-Curcumene ^S^	1483	1483	-	0.10 ± 0.01 ^a^	-	2.11 ± 0.08 ^b^	-	-	-
40.	(*E*)-β-Ionone ^S^	1486	1485	-	1.52 ± 0.09 ^a^	-	1.02 ± 0.05 ^a^	-	0.70 ± 0.03 ^a^	-
41.	Pentadecane ^S^	1500	1500	-	3.81 ± 0.09 ^a^	0.20 ± 0.01 ^b^	3.10 ± 0.11 ^a^	-	0.71 ± 0.03 ^b^	-
42.	Dihydro-actinolide *	1528	1537	-	1.02 ± 0.01 ^a^	-	-	-	-	-
43.	Hexadecane ^S^	1600	1600	-	0.50 ± 0.01 ^a^	-	2.41 ± 0.12 ^b^	-	-	-
44.	Benzophenone ^S^	1627	1625	-	0.32 ± 0.01 ^a^	-	0.80 ± 0.05 ^a^	-	-	-
45.	(*E*)-Hepta-dec-8-ene	1678	1676	-	1.40 ± 0.09 ^a^	-	2.41 ± 0.03 ^b^	0.22 ± 0.01 ^c^	0.71 ± 0.02 ^c^	0.82 ± 0.01 ^c^
46.	Heptadecane ^S^	1700	1700	4.82 ± 0.16 ^a^	41.50 ± 2.01 ^b^	32.51 ± 1.85 ^c^	52.62 ± 2.30 ^d^	23.44 ± 1.01 ^c^	9.41 ± 0.09 ^a^	7.20 ± 0.08 ^a^
47.	Loliolide	1763		-	-	-	-	-	-	3.51 ± 0.08^a^
48.	Octadecane ^S^	1800	1800	-	0.10 ± 0.01 ^a^	-	1.60 ± 0.08 ^a^	-	-	-
49.	Neophyta-diene ^S^	1840	1838	-	-	-	-	-	-	3.20 ± 0.11 ^a^
50.	Hexahydro-farnesyl acetone (Phytone) ^S^	1845	1845	-	-	-	-	1.61 ± 0.09 ^a^	5.91 ± 0.11 ^b^	-
51.	Diisobutyl phthalate ^S^	1867	1868	-	-	-	0.40 ± 0.01 ^a^	2.22 ± 0.12 ^b^	0.82 ± 0.02 ^a^	-
52.	Nonadec-1-ene **	1872	1880	-	0.31 ± 0.01 ^a^	-	0.70 ± 0.02 ^a^	-	0.71 ± 0.03 ^a^	0.70 ± 0.02 ^a^
53.	Hexadecan-1-ol ^S^	1882	1882	-	-	-	-	-	1.21 ± 0.15 ^a^	3.10 ± 0.21 ^b^
54.	Nonadecane ^S^	1900	1900	-	0.10 ± 0.01 ^a^	-	0.81 ± 0.02 ^a^	-	0.31 ± 0.01 ^a^	-
55.	Dibutyl phthalate ^S^	1961	1960	-	-	-	-	9.80 ± 0.15 ^a^	1.03 ± 0.10 ^b^	-
56.	Hexadeca-noic acid ^S^	1963	1960	-	-	-	-	-	-	17.51 ± 1.13 ^a^
57.	Eicosane ^S^	2000	2000	-	0.40 ± 0.01 ^a^	-	-	-	-	-
58.	Cyclooctasulfur	2009	2004	-	-	-	-	0.21 ± 0.01 ^a^	5.12 ± 0.09 ^a^	-
59.	(*Z*)-Octedec-9-en-1-ol ^S^	2060	2060	-	-	-	-	-	-	2.51 ± 0.12 ^a^
60.	Octadecan-1-ol ^S^	2084	2083							2.02 ± 0.12 ^a^
61.	Heneicosane ^S^	2100	2100	-	1.40 ± 0.10 ^a^	-	-	-	-	-
62.	(*E*)-Phytol ^S^	2110	2112	-	-	-	-	3.31 ± 0.09 ^a^	58.42 ± 2.50 ^b^	42.30 ± 2.01 ^c^
63.	(*Z*)-Octadec-9-enoic acid ^S^	2147	2146	-	-	-	-	-	-	3.02 ± 0.09 ^a^
64.	Docosane ^S^	2200	2200	-	-	-	-	13.90 ± 1.28 ^a^	0.42 ± 0.08 ^a^	-
65.	Diisooctyl phthalate ^S^	2274	/	-	-	-	-	13.30 ± 1.11 ^a^	-	-

**I**—HS-SPME (DVB/CAR/PDMS fiber) of fresh *C. bursa* (HS-F); **II**—HS-SPME (DVB/CAR/PDMS fiber) of air-dried *C. bursa* (HS-D); **III**—HS-SPME (PDMS/DVB fiber) of fresh *C. bursa* (HS-F); **IV**—HS-SPME (PDMS/DVB fiber) of air-dried *C. bursa* (HS-D); **V**—hydrodistillate of fresh *C. bursa* (HD-F); **VI**—hydrodistillate of air-dried *C. bursa* (HD-D); **VII**—supercritical CO_2_ extract of freeze-dried *C. bursa* (FD-CB); RI—retention indices relative to C_9_–C_25_ alkanes; RI_L_—retention indices from the literature (NIST Chemistry WebBook, NIST Standard Reference Database Number 69, http://webbook.nist.gov/chemistry/); *—tentatively identified; **—correct isomer is not identified; ^S^—identification confirmed with standard compound; SD—standard deviation; the same upper letter in the same row of analysed variable indicates no significant differences (Duncan’s test, *p* < 0.05).

**Table 2:** Fatty acid content of freeze-dried *C. bursa* (% of total fatty acid content; SD—standard deviation).

No	Fatty Acids	% ± SD
**1.**	Caprylic acid (C8:0)	0.055 ± 0.007
**2.**	Capric acid (C10:0)	0.261 ± 0.006
**3.**	Lauric acid (C12:0)	2.106 ± 0.000
**4.**	Tridecyclic acid (C13:0)	0.373 ± 0.007
**5.**	Myristic acid (C14:0)	2.891 ± 0.028
**6.**	Palmitic acid (C16:0)	25.439 ± 0.050
**7.**	Palmitoleic acid (C16:1)	3.514 ± 0.025
**8.**	Margaric acid (C17:0)	0.372 ± 0.001
**9.**	Stearic acid (C18:0)	9.042 ± 0.009
**10.**	*trans*-oleic acid + *cis*-oleic acid (C18:1n9*t* + C18:1n9*c*)	36.53 ± 0.079
**11.**	Linoleic acid (C18:2n6*c*)	11.619 ± 0.045
**12.**	γ-linolenic acid (C18:3n6)	0.362 ± 0.001
**13.**	α-linolenic acid (C18:3n3)	1.344 ± 0.005
**14.**	Arachidic acid (C20:0)	0.408 ± 0.022
**15.**	Paullinic acid (C20:1)	0.789 ± 0.051
**16.**	Arachidonic acid (C20:4n6)	1.563 ± 0.052
**17.**	Eicosatrienoic acid (C20:3n3)	1.065 ± 0.032
**18.**	Behenic acid (C22:0)	1.365 ± 0.025
**19.**	Nervonic acid (C24:1)	0.887 ± 0.002
Total saturated fatty acids (SFA)		42.32
Total mono-unsaturated fatty acids (MUFA)		41.73
Total poly-unsaturated fatty acids (PUFA)		15.95
Total ω3 fatty acids		2.41
Total ω6 fatty acids		13.54

**Table 3:** Antifungal (MIC_50_) and fungal growth inducing (GIC_50_) effect of tested water (H_2_O) and dimethyl sulfoxide (DMSO) extracts of *C. bursa* on selected fungal species.

Microorganism	MIC_50_		GIC_50_	
	H_2_O Extract	DMSO Extract	H_2_O Extract	DMSO Extract
*Alternaria alternata*	500	500	-	-
*Aspergillus flavus*	-	5000	5	5, 50
*Aspergillus ochraceus*	500	-	-	5000
*Fusarium graminearum*	5000	-	-	-
*Fusarium verticillioides*	500	-	-	-
*Penicillium expansum*	-	50	5000, 500	-
*Rhizophus* spp.	-	5000	5000	-

MIC_50_—minimal inhibitory concentration reducing the optical growth for at least 50% of the tested fungi; GIC_50_—minimal growth inducing concentration resulting in at least 50% increase of growth for the tested fungi; all numbers in Table 3 are expressed as concentrations of used extracts in µg/mL; “-” is used when no significant change was observed.
